# Study on the use and effectiveness of malaria preventive measures reported by employees of Chinese construction companies in Western Africa in 2021

**DOI:** 10.1186/s12889-023-15737-1

**Published:** 2023-05-03

**Authors:** Li Zou, Ke Ning, Wenyu Deng, Xufei Zhang, Mohammad Shahir Sharifi, Junfei Luo, Yin Bai, Xiner Wang, Wenjuan Zhou

**Affiliations:** 1 School of literature and journalism, Central South University, Changsha, Hunan Province China; 2 Insurance Professional College, Changsha, Hunan Province China

**Keywords:** Western Africa, Infrastructure Construction, Chinese Employees, Malaria, Infection Risk

## Abstract

**Background:**

As malaria continues to be a significant global public health concern, especially in Sub-Saharan Africa, Chinese workers in Africa are at increased risk of malaria. The effectiveness of malaria prevention measures implemented by Chinese companies and workers is a question that may correlate with the malaria infection rate in this population. This study explored the use and effectiveness of malaria prevention measures for Chinese employees in West Africa to provide a reference for companies and individuals on improving malaria prevention and control.

**Methods:**

Using a cross-sectional approach, we surveyed 256 participants in 2021, mainly from Nigeria, Mali, Côte d’Ivoire, Ghana, Guinea, Sierra Leone, and Senegal in West Africa. The survey duration is from July to the end of September 2021. We selected two companies from the 2020 ENR "World’s Largest 250 International Contractors" list, which featured 6 Chinese companies, all of which are state-owned and have a 61.9% market share in Africa. The participants were Chinese workers with more than a year of work experience in construction companies in Africa. A 20-minute WeChat-based structured online questionnaire was used to obtain information on malaria infection status and malaria prevention measures. Descriptive statistical analysis, chi-square test, principal components analysis, and ordinal logistic regression analysis are used to analyze the data obtained. The difference in Statistical significance was set at P < 0.05.

**Results:**

Ninety six (37.5%) participants contracted malaria more than once within a year. The principal components analysis found a low correlation between public and individual preventive measures. No significant correlation was found between public preventive measures and malaria infection (p > 0.05), while standardized use of mosquito nets (P = 0.016) and pesticide spraying (P = 0.047) contributed significantly to fewer malaria infections at the individual level, but the removal of vegetation around houses (P = 0.028) at the individual level related to higher malaria infection.

**Conclusions:**

In our sample of Chinese construction workers going to Africa, some individual preventive measures had a stronger association with malaria prevention than a variety of public environmental measures. Furthermore, individual and public preventive measures were not associated with each other. Both of these findings are surprising and require further investigation in larger and more diverse samples. This- study provides important clues about the challenges that risk reduction programs face for migrant workers from China and elsewhere.

## Introduction

Malaria is one of the three most widespread infectious diseases worldwide. A 90% reduction in malaria incidence and mortality from 2015 to 2030 has been included in the World Health Organization’s Sustainable Development Goals (SDGs) [1]. According to the World Health Organization, there were an estimated 229 million malaria cases globally in 2019, with an estimated 409,000 deaths, most of them in Africa [2].

Malaria is a chronic problem in West Africa and has a major negative impact on economic development. According to annual reported global malaria cases, among the ten African countries with the highest malaria burden from 2010 to 2019 were six West African countries, namely Burkina Faso, Cameroon, Ghana, Mali, Niger, and Nigeria [3]. According to the World Health Organization’s World Malaria Report 2021, countries in West Africa account for 51.1% of malaria cases and deaths globally. Four countries accounted for just over half of the world’s malaria deaths, with Nigeria accounting for 31.9% [4]. Despite intensified malaria control strategies over the past decade, malaria is still prevalent in this region. The economic losses and morbidity burden caused by malaria are still substantial.

Investment in infrastructure construction in sub-Saharan Africa has increased in recent years, and many Chinese engineering companies and workers have carried out infrastructure construction in this region. The Chinese Department of Commercial Affairs reports that China sent 73,200 Chinese workers working in various industries to Africa in 2019, and by the end of the year, there were 182,700 Chinese workers in the region [5]. In 2019, China made its largest investment in the construction industry in sub-Saharan Africa [6].

Most Chinese people have less knowledge of malaria risks and preventive measures. On June 30, 2021, the World Health Organization issued China malaria-free certification. There were no local cases of malaria in China for many years [7]. Ultimately, long-term lack of exposure to malaria led to insufficient knowledge of malaria risks and preventive measures among Chinese workers. Therefore, Chinese workers in areas with high malaria prevalence are likely to face a higher threat of malaria infection due to a lack of malaria awareness. According to statistics, from 2010 to 2019, China reported 37,047 malaria cases, of which 29,248 cases were imported, accounting for 78.95% of all cases [8]. In 2019, China reported 2,674 malaria cases, including 2,673 imported cases [9]. These malaria cases were mainly migrant workers returning to China from West Africa. Four of the top five countries by the number of imported cases are in sub-Saharan Africa: Nigeria, Congo (DRC), Cote d’Ivoire, and Guinea [9]. As China moves towards globalization, the number of migrants traveling between China and West Africa continues to increase. Therefore, it is necessary to investigate the use and effectiveness of malaria prevention measures by Chinese workers in West Africa.

The management team of Chinese companies in West Africa is responsible for implementing, monitoring, and estimating malaria prevention measures at the public level [10]. These measures include the distribution of free mosquito nets, construction of dormitories with anti-mosquito facilities, the cleanliness and hygiene of the environment, etc. As for the measures at an individual level, the project management team reminds the employees that they are personally responsible for the implementation of measures, including the standardization of the use of mosquito nets, lighting mosquito coils, wearing long-sleeved shirts and trousers/skirts, etc. [11]. Management does not enforce adherence at the individual level [10], [11].

In areas of intense malaria transmission, the use of insecticide-treated nets (ITN) has been demonstrated to considerably reduce malaria disease incidence. In addition, the use of ITNs is identified by WHO as one of the main interventions to reduce the burden of malaria [12]. According to the Millennium Development Goals (MDGs) in 2010, a high (80%) coverage of the above protection methods aimed at protecting young children and pregnant women (less than 20% of the population) [13]. According to Bhatt, who conducted a study on the effect of malaria control on Plasmodium falciparum in Africa between 2000 and 2015, the targeted population for malaria protection is primarily women and children, as they are more vulnerable to malaria infection and death, and men are not in their considerable attention [14]. Chinese workers working in Africa are primarily adult men, so local prevention measures and controls do not include them. On the other hand, migrant workers are one of the groups at high risk of malaria. Therefore, travelers and migrants who visit malaria areas for 2–3 months are advised to take some medicines weekly, such as Mefloquine, in a dosage of 5 mg/kg drug for chemoprophylaxis for non-immune travelers [15]. Chinese migrant workers stay in Africa for at least one year, longer than the drug-recommended period. In addition, the local malaria prevention programs with the governments’ financial supports mainly cover local residents, while migrants and travellers are receiving guidance and recommendations for their malaria prevention. Therefore, the Chinese workers and their companies must take responsibility for malaria control themselves.

Chinese workers engaged in a variety of construction jobs in Africa are at severe malaria infection risk. At the same time, local African public health resources consistently did not cover this population. In addition, due to the great distance from mainland China, Chinese public health resources and scholars do not pay much attention to this problem. This study investigates the use and effectiveness of malaria preventive measures implemented at public and individual levels and their relations with malaria infection in infrastructure construction projects operated by Chinese companies in West Africa. It also provides suggestions for improving the effectiveness of malaria prevention in overseas projects and appropriate health interventions for the future.

## Methods

### Study design and sample

This study is a cross-sectional design focusing on the use and effectiveness of malaria prevention measures in infrastructure construction projects run by Chinese companies in West Africa. The data was collected between July and the end of September 2021. The study adopted a cluster sampling strategy. According to the 2020 ENR “World’s Largest 250 International Contractors” List, six Chinese companies, all state-owned, are leading the African market with a 61.9% market share [16]. They mostly use the same management standard and implement the same malaria preventive measures. Although the mentioned companies win construction contracts individually, they then cooperate in implementing the task. In this study, we randomly selected the two companies as a bridge to the research subjects. The companies gave us ways to access respondents who are related to these two companies but may also come from different industries. The criteria for inclusion were that the participants must be Chinese, able to read Chinese, be currently working on the selected projects, have more than one year of work experience in West Africa, and have WeChat on their smartphones. We used G*power software to calculate the sample size. According to the literature review requirements and the multivariate analysis, a total of 25 independent variables had to be included in the analysis, and the effect value was determined to be 0.15, α = 0.05, 1-β = 0.95. The above content was entered into the box, and the total sample size obtained was 252. Considering the non-response rate of 20–30% for the online survey, the minimum sample size in this study is 330 cases. The investigation finally included 334 participants. After informed consent, 256 participants completed a 20-minute online survey using questionnaires obtained on WeChat. This study used WeChat, as it is used by more than 90% of Chinese people, and they are pretty familiar with using it [17]. To avoid data duplication, we declined to accept respondents with the same IP address.

To maintain data quality in this online survey, we first carefully designed the questions to be relevant to the study questions. Second, we allowed participants to choose their answers from multiple graded options. Third, we used several attention-check methods to verify respondents’ diligence or negligence in filling out the survey form. For example, we place some questions twice in different positions of the questionnaire, shifting the position of the answers, asking them to choose the exact answer already given in the question, and using two questions with the same objective in both affirmative and negative phrasing.

### Measuring tools

The survey consists of four parts: The first and second parts contain information on socio-demographics and malaria infection within one year, such as gender, age group, marital status, level of education, occupation, work experience in Africa, and whether or not they had parasite confirmed diagnosed malaria within one year, and if so, how many times. We also asked them whether they had received any treatment for malaria. Part three assesses malaria public preventive measures reported by employees, such as: whether or not the company provides free mosquito nets if they live in a dormitory with a bathroom if they live in a single-person dormitory, do they have a clean and sanitary environment, is the vegetation controlled camp-wide, and the type of housing provided (prefab houses or ship container renovated houses, brick house, villa or apartment). Part four includes individual preventive measures implemented by employees, such as (whether or not they) use mosquito nets according to standard every night, lighting mosquito coils every day, wearing long-sleeved shirts and trousers/skirts while going outside, don’t save water indoors, cleaning up the vegetation around their dwelling every month, and spray insecticide indoors every day. The participants answered the questions in 5 levels - always, often, sometimes, rarely, and never. We categorized the first two levels as yes and the last three as no. The questionnaires used in this survey were adapted from a study on the Malaria Epidemiology Survey among Chinese returnees and international travelers [18], [19].

### Statistical analysis

The data was stored in the Tencent (WeChat operator) questionnaire online repository, and the results were downloaded to SPSS 26 for statistical analysis. Statistical analysis was performed to describe the characteristics of the participants. A chi-square test was launched to test the correlation of malaria infection within one year with individual and public preventive measures. Principal components analysis was used to test the correlation between individual and public preventive measures. Ordinal logistic regression analysis was used to determine whether the relevant variables independently affected malaria infection within one year. We used ordinal regression for the three categories: 1 = no infections, 2 = one infection, and 3 = 2 to 5 infections. Since individual and public measures are not associated with our sample, we performed two separate regression analyses. First, malaria infection within one year was the dependent variable, and individual preventive measures were independent. Second, with the same dependent variable, we included all public preventive measures as independent variables. The difference in Statistical significance was set at P < 0.05. We also performed a principal component analysis of the correlation matrix of twelve risk variables for malaria.

## Results

### Respondent characteristics and malaria infection rate reported by respondents within one year

Of the 334 Chinese employees invited, 256 took part in the survey. The majority were men (96.1%, n = 246), aged 18–60 years (100.0%), and married (70.7%). About 12.5% received a junior high school education, 26.6% received a high school or high vocational education, and 60.9% received a junior college or higher education. Among the jobs, labor workers accounted for 9.0%, office clerks 5.5%, administrators 63.7%, technicians 16.8%, and logistics 3.1%. Nearly 39.4% of the participants have worked in Africa for 1 to 2 years, 18.8% have worked in Africa for 3 to 5 years, and 41.8% have worked in Africa for more than five years. We found that 37.5% of the respondents had been infected with malaria more than once within one year (Table 1).

**Table 1:** General demographic characteristics and six-month malaria prevalence (n = 256)

Item	Category	Number	Percentage
Gender			
	male	246	96.1
	female	10	3.9
Age-group			
	18–30	75	29.3
	31–40	92	35.9
	41–50	67	26.2
	51–60	22	8.6
Marital status			
	Single	73	28.5
	Married	181	70.7
	Divorced	2	0.8
Education Level			
	Junior High School	32	12.5
	High School or Vocational High	68	26.6
	Junior College or Above	156	60.9
Jobs			
	Labors	23	9.0
	Office Clerk	14	5.5
	Site Administrator	163	63.7
	Technician	43	16.8
	Logistics	8	3.1
	Other	5	2.0
Working experience in Africa			
	1 ~ 2 Years	101	39.4
	3 ~ 5 Years	48	18.8
	5 Years Above	107	41.8
Infected with malaria within one year			
	no infections	160	62.5
	one infection	57	22.3
	2 to 5 infections	39	15.2

### Public preventive measures and individual preventive measures for malaria

The survey findings show that 179 people (69.9%) received free mosquito nets from their companies, 169 people (66.0%) used dorms with a bathroom, 156 people (60.1%) lived in single-person dormitories, 200 people (78.1%) lived in a clean and sanitary environment, 15 people (5.9%) reported that vegetation in the project camp was effectively cleaned up, 52 people (22.2%) lived in villas or apartments, 64 people (25.0%) lived in prefab houses or renovated container houses (Table 2).

**Table 2:** Characteristics of public preventive measures and individual preventive measures (n = 256)

	Item	Category	Number	Percentage
*Public level*	*Free distribution of mosquito nets by company*	Yes	179	69.9
		No	77	30.1
	*Live in a dormitory with a bathroom*	Yes	169	66.0
		No	87	34.0
	*Live in a single-person dormitory*	Yes	157	61.3
		No	99	38.7
	*Live in a clean and sanitary living environment*	Yes	200	78.1
		No	56	21.9
	*Vegetation effectively controlled camp-wide*	Yes	15	5.9
		No	241	94.1
	*Housing type*	Villa or Apartment	52	20.3
		Brick house	117	45.7
		Prefab houses/Ship container renovated houses	64	25.0
Individual level	Standardized use of mosquito nets every night	Yes	135	52.7
		No	92	35.9
	Lighting mosquito coils every day	Yes	127	49.6
		No	129	50.4
	Wearing long-sleeved shirts and trousers/skirts while going outside	Yes	172	67.2
		No	84	32.8
	Don’t save water indoors	Yes	56	21.9
		No	200	78.1
	Clean up the vegetation around the house every month	Yes	92	35.9
		No	164	64.1
	Spray insecticide indoor every day	Yes	153	59.8
		No	103	40.2

In addition to the public preventive measures implemented by the project management team, the employees also implemented individual preventive measures. Individual preventive measures commonly include standard use of mosquito nets (59.5%), lighting mosquito coils (49.6%), wearing long-sleeved shirts and trousers/skirts (67.2%), don’t save water indoors (21.9%), cleaning up vegetation around the self-owned house (35.9%) (Table 2).

### Correlation analysis between public and individual preventive measures

There are six public preventive measures and six individual preventive measures. The unrotated two-factor solution, which accounts for approximately 40% of the total variance, is presented in Table 3. The first factor is dominated by individual preventive measures, all of which have six positive factor loadings. Four of the public preventive measures are positively associated with the second factor, while two of them, general clean-up of the vegetation around the camp and distribution of free mosquito nets, are negatively associated.

**Table 3:** Factor analysis of public and individual preventive measures

	Item	Component 1	Component 2
*Public level*	*Vegetation effectively controlled camp-wide*	-0.174	-0.260
	*Live in a clean and sanitary living environment*	0.120	0.564
	*Live in a single-person dormitory*	-0.138	0.530
	*Live in a dormitory with a bathroom*	0.234	0.303
	*Free distribution of mosquito nets by company*	0.154	-0.442
	*Housing type*	-0.022	0.641
Individual level	Standardized use of mosquito nets every night	0.395	0.107
	Lighting mosquito coils every day	0.528	-0.237
	Wearing long-sleeved shirts and trousers/skirts while going outside	0.655	-0.006
	Don’t save water indoors	0.658	0.027
	Clean up the vegetation around the house every month	0.702	-0.081
	Spray insecticide indoors every day	0.644	0.151

### Analysis of related factors of malaria infection within one year

Using ordinal regression, we found that two of the individual measures were negatively associated with our ordinal measure of infection frequency over the past year: standardized use of mosquito nets (P = 0.016) and spray insecticide (P = 0.047) (Table 4). Cleaning up the vegetation around the self-owned house had a positive association (P = 0.028) with malaria infection. The public preventive measures were not statistically associated with a malaria infection within one year among the employees (Table 5).

**Table 4:** Multivariate ordinal logistic regression analysis of individual preventive measures and malaria infection

Variable	Ordinal Regression coefficient(95%CI)	P-Value
Standardized use of mosquito nets every night (yes = 1, no = 0)	-0.686 (-1.245, -0.128)	0.016
Lighting mosquito coils every day (yes = 1, no = 0)	-0.237 (-0.819, 0.345)	0.425
Wearing long-sleeved shirts and trousers/skirts while going outside (yes = 1, no = 0)	0.365 (-0.285, 1.015)	0.271
Don’t save water indoors (yes = 1, no = 0)	-0.444 (-1.198, 0.311)	0.249
Clean up the vegetation around the house every month (yes = 1, no = 0)	0.706 (0.077, 1.336)	0.028
Spray insecticide indoors every day (yes = 1, no = 0)	-0.638 (-1.267, -0.009)	0.047

1 = never or not in last year; 2 = once in last year; 3 = 2 to 5 times in last year

**Table 5:** Multivariate ordinal logistic regression analysis of public preventive measures and malaria infection

Variable			Ordinal Regression coefficient(95%CI)	P-Value
Free distribution of mosquito nets by company (yes = 1, no = 0)			-0.122 (-0.728, 0.485)	0.694
Live in a dormitory with a bathroom (yes = 1, no = 0)			-0.283 (-0.858,0.292)	0.334
Live in a single-person dormitory (yes = 1, no = 0)			0.061 (-0.501, 0.623)	0.831
*Live in a clean and sanitary living environment* (yes = 1, no = 0)			-0.532(-1.175, 0.112)	0.105
*Vegetation effectively controlled camp-wide* (yes = 1, no = 0)			0.406 (-0.694, 1.506)	0.469
Housing type (Prefab houses/Ship container renovated houses = 2, Brick house = 1, Villa or Apartment = 0)			-0.382 (-1.192, 0.428) -0.319(-0.954, 0.317)	0.356 0.325

1 = never or not in last year; 2 = once in last year; 3 = 2 to 5 times in last year

## Discussion and conclusion

This study is the first cross-sectional study aimed at describing the association between malaria infection and the use of malaria preventive measures among the Chinese infrastructure construction companies in West Africa. We found that 37.5% of participants had had malaria infection in the past year, and many had had malaria infection more than once. In the African region, for instance, in Nigeria, the 2021 Nigeria Malaria Indicator Survey Report has shown that the malaria prevalence in the country was 22% [20]. However, what we measured was the malaria infection rate reported by our respondents, while it was malaria prevalence data of the African region, therefore, the two figures are incomparable. This finding suggests that Chinese infrastructure companies in West Africa are at high risk of malaria as more and more Chinese workers travel between China and Africa [21], [22]. A better understanding of the use and effectiveness of malaria preventive measures among Chinese workers in West Africa supports interventions to reduce malaria infection rates and improve the effectiveness of preventive measures. The study provides important clues about the challenges that risk reduction programs face for migrant workers from China and elsewhere.

Neither the local malaria program in Africa nor the domestic malaria prevention and control resources in China covers the Chinese infrastructure workers going to Africa. Local aid in Africa mainly targets key groups of citizens [14]. China’s Department of Disease Control distributes prevention manuals to outbound centers, but it is primarily the responsibility of employing companies to support prevention measures [8], [10].

Prevention at the public level is managed by company officials and consists of the free distribution of mosquito nets, construction of dormitories with anti-mosquito facilities, and maintenance of public environment cleanliness. Workers’ responsibilities for prevention consist of strategies such as wearing long sleeves, use of mosquito nets every night, use of mosquito coils, and maintenance of private living spaces [23]. Our research found correlations within the sphere of public prevention measures and prevention measures undertaken by individual workers. However, there is a lack of correlations between the public and individual parties. This finding is consistent with reports that malaria prevention and control measures in China’s overseas infrastructure projects are carried out separately by different responsible persons who do not usually coordinate with each other [11]. The disconnect between the two forms of prevention evident in our small study should be investigated in larger and more diverse samples of migrant workers. What are the currently missed opportunities to connect prevention aimed at the community environmental level with motivation among workers to protect themselves?

We found that individuals who adhere to the standardized use of mosquito nets and spray insecticide indoors have a lower malaria infection rate within one year. Cleaning up the vegetation around the self-owned house predicts a higher number of infections. The reason for this could be that cleaning vegetation itself may pose a risk of malaria infection, exposing those who do it to mosquito bites. Several of the individual behaviors we hypothesized as protective measures—covering arms and legs, lighting mosquito coils, and avoiding water containers indoors --- did not show significant associations with infection rates. It is possible that such measures have only small additional effects on infection, given the use of nets and spraying. However, the potential of these behaviors for significantly reducing risk when rigorously applied should be investigated in studies with better measures of compliance.

The apparent absence of association between infection risk and management-initiated practices that potentially have community-wide effects requires explanation. Although what we have termed public measures are based on worker self-reports, they are responses to simple questions that workers should be able to answer accurately. The finding that free distribution of mosquito nets is not correlated with infection rates but worker use every night should alert us to the fact that free resources have to be used regularly to be effective. It is not that good housing, and a clean environment are unimportant, but they must be used effectively by workers who are motivated to keep free from malaria.

To enhance the connection between what management attempts to do with housing and the environment and what workers do on their own to limit their infection risks may require a different approach. The project management team is responsible for implementing public precautions, but their main job is to deal with construction affairs. Malaria prevention is a long and meticulous task that requires specialized professionals. Increased management attention and participation of professionals in implementing public preventive measures may result in greater sustainability of malaria control effectiveness [24]. However, there are many barriers to overcome to realize this possibility. First of all, in China’s infrastructure projects in Africa, public measures for malaria prevention and control are not implemented by public health professionals and institutions but by company management departments following disease control recommendations. Secondly, the companies did not set up a special department responsible for implementing, supervising, and evaluating public measures. These were carried out part-time by the safety and environmental protection staff. Finally, malaria infection rates in these establishments were not used as an indicator to revise malaria control measures.

This finding suggests that Chinese infrastructure companies going to Africa should connect management-initiated malaria prevention and environmental measures with enhanced efforts to motivate workers on their own behalf. The focus of prevention and control should include improving individual worker awareness of prevention and control.

## Limitations

The study’s primary limitation was that the samples came from a subset of countries in West Africa, participants’ recall bias was likely to influence the study’s internal validity, and due to an online survey design, the 20% non-response rate could affect the results. Therefore, more adequate sample sizes to represent the region are needed to provide better and more general findings. Given the widespread concerns about malaria infection rates and the use of prevention measures, the results of this study can serve as a baseline for improving malaria control at Chinese infrastructure companies in West Africa.

## Data Availability

The datasets used and analyzed during the current study are available from the corresponding author on reasonable request.
