# Correction: Study on the use and effectiveness of malaria preventive measures reported by employees of Chinese construction companies in Western Africa in 2021

**DOI:** 10.1186/s12889-023-17105-5

**Published:** 2023-11-02

**Authors:** Li Zou, Ke Ning, Wenyu Deng, Xufei Zhang, Mohammad Shahir Sharifi, Junfei Luo, Yin Bai, Xiner Wang, Wenjuan Zhou

**Affiliations:** 1 School of literature and journalism, Central South University, Changsha, Hunan Province China; 2 Insurance Professional College, Changsha, Hunan Province China

**BMC Public Health (2023) 23:813**

10.1186/s12889-023-15737-1

The original publication of this article contained an incorrect author name. The incorrect and correct information is listed in this correction article. The original article has been updated.

Incorrect

Mohamamad Shahir Sharifi

Correct

Mohammad Shahir Sharifi

